# Motion Correction of Whole-Body PET Data with a Joint PET-MRI Registration Functional

**DOI:** 10.1186/1475-925X-13-S1-S2

**Published:** 2014-02-28

**Authors:** Michael Fieseler, Fabian Gigengack, Xiaoyi Jiang, Klaus P Schäfers

**Affiliations:** 1 European Institute for Molecular Imaging, University of Münster, Mendelstrasse 11, 48149 Münster, Germany; 2 Department of Computer Science, University of Münster, Einsteinstrasse 62, 48149 Münster, Germany

**Keywords:** Motion Correction, PET-MRI, Image Registration, Joint Registration, Multi-Modal Data, Positron Emission Tomography, Magnetic Resonance Imaging

## Abstract

Respiratory motion is known to degrade image quality in PET imaging. The necessary acquisition time of several minutes per bed position will inevitably lead to a blurring effect due to organ motion. A lot of research has been done with regards to motion correction of PET data. As full-body PET-MRI became available recently, the anatomical data provided by MRI is a promising source of motion information. Current PET-MRI-based motion correction approaches, however, do not take into account the available information provided by PET data. PET data, though, may add valuable additional information to increase motion estimation robustness and precision.

In this work we propose a registration functional that is capable of performing motion detection in gated data of two modalities simultaneously. Evaluation is performed using phantom data. We demonstrate that performing a joint registration of both modalities does improve registration accuracy and PET image quality.

## Introduction

Respiratory motion is known to impair image quality as well as quantification in positron emission tomography (PET) [1]. As the acquisition of PET takes several minutes per bed position, organ motion due to respiration cannot be avoided and will thus result in blurred images. By using gating methods, the acquired PET data can be divided into different motion phases. Gating reduces the amount of motion contained within each gate to a large extent, yet at the expense of reduced statistics and thereby image quality [2,3]. To alleviate this, motion between gates can be estimated and subsequently be used to correct PET data for motion, resulting in a single image volume with reduced motion artifacts and full statistics. Various approaches for motion estimation of gated PET data have been studied, including optical flow [4], B-spline based methods [5], and registration methods including mass-preservation [6].

The use of 4D-CT data for motion correction has been proposed [7]. The advantage of this approach lies in the usage of anatomical data, which is independent of tracer uptake. Acquisition of 4D-CT data, however, increases the radiation burden for the patient.

Whole body PET-MRI is promising regarding PET motion correction. High resolution MR data may allow for a precise motion estimation independent of tracer uptake and without additional radiation burden for the patient. The feasibility of PET-MRI-based motion correction of PET data has been demonstrated already, e.g., using hardware phantoms [8], animals [9], and simulation data [10]. A commonality of current approaches to MR-based motion correction is that a 4D MR dataset is acquired from which motion is estimated and subsequently used in the correction of PET data. Strategies for the generation of 4D MR include acquisition of 2D slices with subsequent reordering [11], fast, consecutive acquisitions of 3D volumes [12], and sorting of k-space data during or after acquisition [13,14]. Fayad et al. propose an approach where motion and image data are estimated simultaneously from the acquired MR data [15].

The motion information contained in PET data, however, remains unused in these approaches. In clinical routine, time for the acquisition of MR data needed for motion correction may be limited, as clinical protocols may demand for further, diagnostic sequences [16]. Since time is proportional to image quality in MR, limited acquisition time may not allow to exploit the full potential of MR, resulting in poorer image quality than technically possible. Accordingly, all available information for motion estimation, including PET data, should be used.

Further, both modalities may contribute valuable information to the motion detection process. In MR, e.g., the lungs give relatively little signal due to their low proton density [14]. Integrating information from PET data, if, e.g., active lesions are present in the lungs, may benefit motion estimation. Using information from both modalities should result in more reliable registration results. In the present work we propose an approach that uses information from both modalities by combining two registration functionals into a joint functional.

## Methods

For motion correction approaches in PET-MRI, proper synchronisation of MR and PET is mandatory. The motion determined from MR has to be related to the PET data with respect to time. For the approach described in the following, we assume a gated PET dataset as well as a series of MR datasets. We assume that each PET gate corresponds to one MR gate with respect to its motion phase. The feasibility of creating corresponding phases has been demonstrated, e.g., in [14].

In the following we describe the proposed registration functional, followed by a description of the phantom data used in this work.

### Registration Functional

Registration can be formulated as the problem of finding the transformation *y *that minimizes

(1) J(y)=D(T(y),R)+α⋅S(y)

where  is a distance functional, is the reference volume,  is the template volume to be registered, and  is a regularizer penalizing unfavourable transformations. The scalar value *α *weights the influence of the regularizer. For non-rigid transformations, *y *is chosen as a non-parametric transformation (one vector per voxel).

The registration functional J(y) in Equation (1) could be applied to each modality independently. Ideally, the resulting transformations for both modalities, PET and MR, should be equal. This, however, will not occur in reality, as both modalities do not provide the same, but complimentary information. The objective is thus to combine two registration functionals into one:

(2) $$J\left(y\right)=D\left(T_{\text{MR}}\left(y\right),R_{\text{MR}}\right)+\beta \cdot \;D\left(T_{\text{PET}}\left(y\right),R_{\text{PET}}\right)+\alpha \;\cdot \;S\left(y\right)$$

Here, $R_{\text{MR}}$ and $R_{\text{PET}}$ denote two reference volumes and $T_{\text{MR}}$ and $T_{\text{PET}}$ the template volumes. The scalar value *β *allows to weight the influence of the data term for PET.

In the registration functional in Equation (2), the deformation is represented by a common grid *y *for both modalities. Since the input data will not necessarily share the same resolution, resampling to a common grid is performed. Here, we chose a common grid of 2 mm^3 ^voxel size.

For  we use a hyperelastic regularizer for its ability to penalize changes in volume, area, and length separately. The hyperelastic regularizer $S^{\text{hyper}}$ is defined as

(3) $$S^{\text{hyper}}\left(y\right)=\alpha_{l}\cdot S^{\text{length}}\left(y-y^{\text{ref}}\right)+\alpha_{a}\cdot S^{\text{area}}\left(y\right)+\alpha_{v}\cdot S^{\text{volume}}\left(y\right),$$

where *y*^ref ^is a reference grid, given by the identity transformation in our case. The three summands control changes in length, surface area, and volume. Parameters for the hyperelastic regularizer were chosen empirically as *α_l _*= 1, *α_a _*= 0.1, *α_v _*= 1. Throughout all experiments in this paper, we keep *α_l_*, *α_a_*, *α_v _*fixed and vary the regularization strength by changing *α *in Eq. (2). For further details regarding the regularizer we refer to [17].

As the distance functional , we choose the sum of squared differences (SSD) for both PET and MR. The functional was implemented using the Matlab-based FAIR toolbox [18]. For all registration experiments, linear interpolation was used and a multi-level approach using a downscaling factor of 0.5 was applied. Optimiziation was performed using a Gauss-Newton scheme with a preconditioned conjugate gradient solver.

### Phantom Data

In the present work we use data generated using a software phantom for evaluation. This allows us to compare motion estimates against ground-truth motion data. The XCAT phantom [19] is widely used as the basis for the simulation of imaging modalities and the evaluation of correction methods, e.g., in [6,20,21]. Using the XCAT phantom, we created an artificial PET-MRI dataset as described in the following.

The XCAT phantom was set to a maximum diaphragm motion of 2 cm. We selected eight frames representing the full range from inspiration to expiration [22].

For the creation of MR data a labelled XCAT dataset of 1 mm^3 ^resolution was created. This dataset and known tissue values for *T*_1_, *T*_2_, and proton density [23,24] were used as an input to the freely available MR simulation software SIMRI [25]. We simulated an MR acquisition of stacked 2D slices covering the thorax. For respiratory motion, the largest amount of motion can be expected in the cranio-caudal and anterior-posterior directions. Thus, a sagittal slice orientation was chosen to capture these directions in-plane. For the phantom dataset used here, the maximum extents of motion are 2.5 mm (left-right), 10.9 mm (anterior-posterior), and 25.2 mm (head-feet). The following MR parameters were used: gradient echo, TE/TR 10 ms/30 ms, 12° flip angle, 256 × 256 pixels, 2 mm pixel size in-plane, slice spacing 1 cm, slice thickness 1 cm.

**Figure 1:**
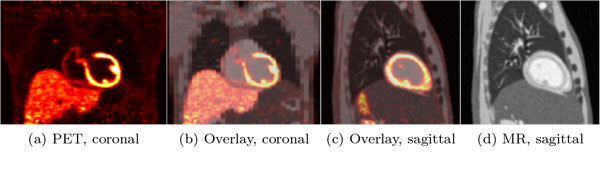
**Phantom Data (a) Simulated PET data, coronal view**. Visible are lesions in both lungs and in the liver. (b) Overlay of PET and MR, coronal view. (c) Overlay of PET and MR, sagittal view. (d) Simulated MR data, sagittal slice.

For PET data creation an XCAT dataset with lesions added to the lungs and the liver was created (heart 50 kBq/ml, liver 20 kBq/ml, background 2 kBq/ml, lesions 50 kBq/ml, lesion diameter 5 mm). Sinograms were created by forward-projection using the geometry of the Siemens Biograph mMR scanner. Poisson noise was added to the sinograms. Additionally, attenuation was added using the attenuation maps provided by the XCAT phantom. For comparison, a second phantom as described above was created, but without lesions. Subsequently, the sinograms were reconstructed. All projections and reconstructions were performed using EMRECON[26]. The generated data is shown in Figure 1.

### Evaluation

Using the phantom data described above allows for evaluation based on ground-truth motion as well as ground-truth activity data. First, we set *β *= 0, thereby performing an MR-based registration and use varying values of *α *to determine the best result for an MR-based registration.

Using the determined registration parameters, we compute registrations for increasing values of *β*, thus adding increasing amounts of PET information to the registration.

The resulting deformation fields *y *are compared to the ground-truth motion provided by the XCAT phantom by means of the *averaged endpoint error *(AEE) defined by

$$\text{AEE(}y\text{,}\;y^{\text{GT}}\text{)}\;\text{ = }\;\frac{1}{\left|\Omega \right|}\cdot \underset{x\in \Omega }{\sum }\sqrt{\overset{3}{\underset{i=1}{\sum }}{\left(y_{i}\left(x\right)-y_{i}^{\text{GT}}\left(x\right)\right)}^{2}}$$

where Ω is the image domain, *y_i _*the i-th component of vector *y*, and *y*^GT ^the ground-truth vector. Here, we examine averaged values of the AEE for all gates.

Further, the computed motion estimates are used to perform a motion correction of the dataset. The PET gates are warped using the computed motion and averaged. We evaluate correlation values of the corrected images as well as the recovered activities in the three lesion regions. For all registrations, attenuated PET data are used. For evaluation of correlation coefficients and activity recovery, attenuation corrected PET data are used.

## Results

### Registration Accuracy

**Figure 2:**
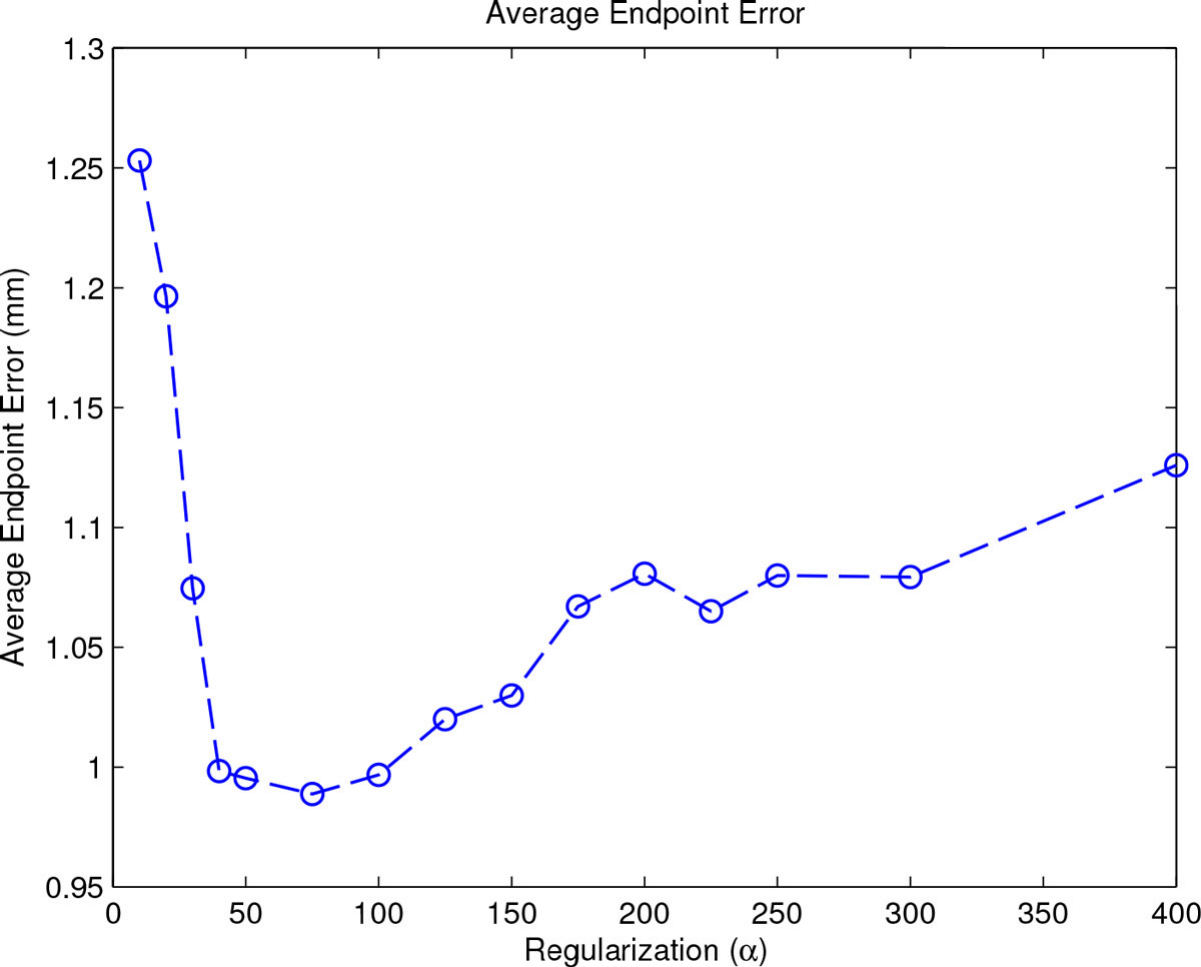
**Average Endpoint Error for MR-only registration**. The figure shows the global average endpoint error (AEE) for a range of regularization values *α*. PET weight *β *is kept to zero here. A minimum at *α *= 75 is observable.

Figure 2 shows the average endpoint error for registrations with increasing regularization strength *α*. A value of *α *= 75 minimizes the overall average endpoint error.

**Figure 3:**
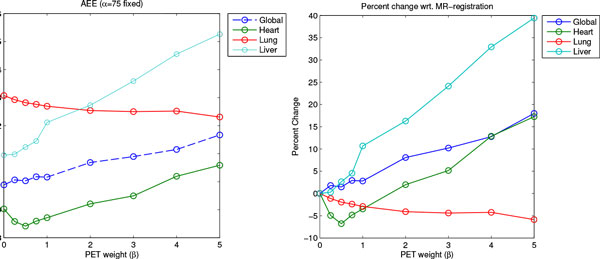
**Average Endpoint Error for varying degrees of PET influence**. Shown is the AEE for local regions and globally for increasing additions of PET to the registration (weight *β*). The upper plot gives the AEE, the lower plot the percentage change with respect to the MR-only registration (*α *= 75, *β *= 0).

Figure 3 shows results for increasing values of *β*. By increasing *β*, AEEs for the heart and the lung are slightly reduced, whereas the global error and the error in the liver region increases slightly.

**Figure 4:**
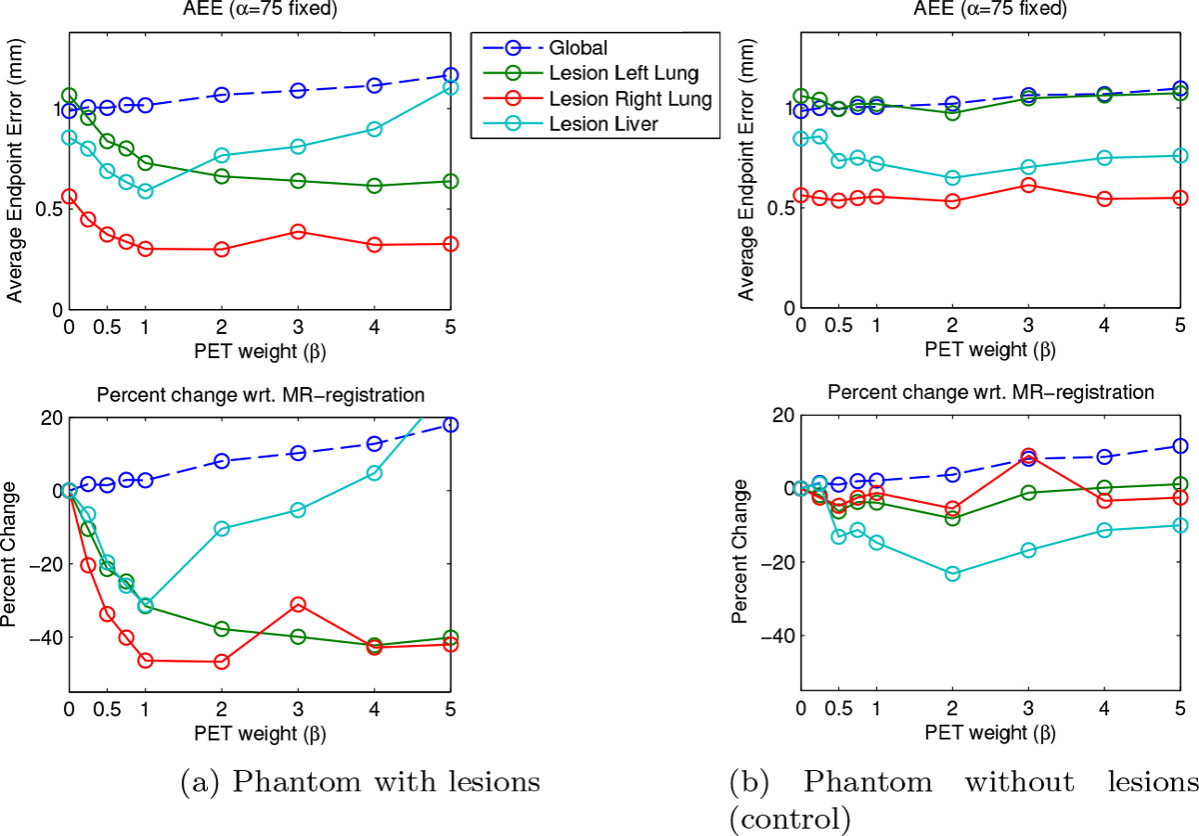
**AEE for lesions**. (a) The upper plot shows the AEE for different weights *β*. A large reduction of AEE with increasing weight *β *is observable for the lesion regions. (b) Same evaluation as in (a) but for registrations using a phantom *without *lesions. The reduction of average endpoint error is more pronounced when active lesions are present, indicating that it is the information by the lesions that leads to an improvement.

In Figure 4 AEEs for the lesion regions are shown. In Figure 4a we show results for the phantom with added lesions as described above. For all three lesion regions a significant decrease in AEE can be observed. To examine if the decrease in AEE is indeed caused by a better registration due to the information provided by the lesions, we repeated this experiment with the same phantom but without lesions. Results for this experiment are shown in Figure 4b. Apart from the liver lesion region, the AEE for the lesions remains almost unchanged for increasing values of *β*.

### Correlation Coefficients

**Figure 5:**
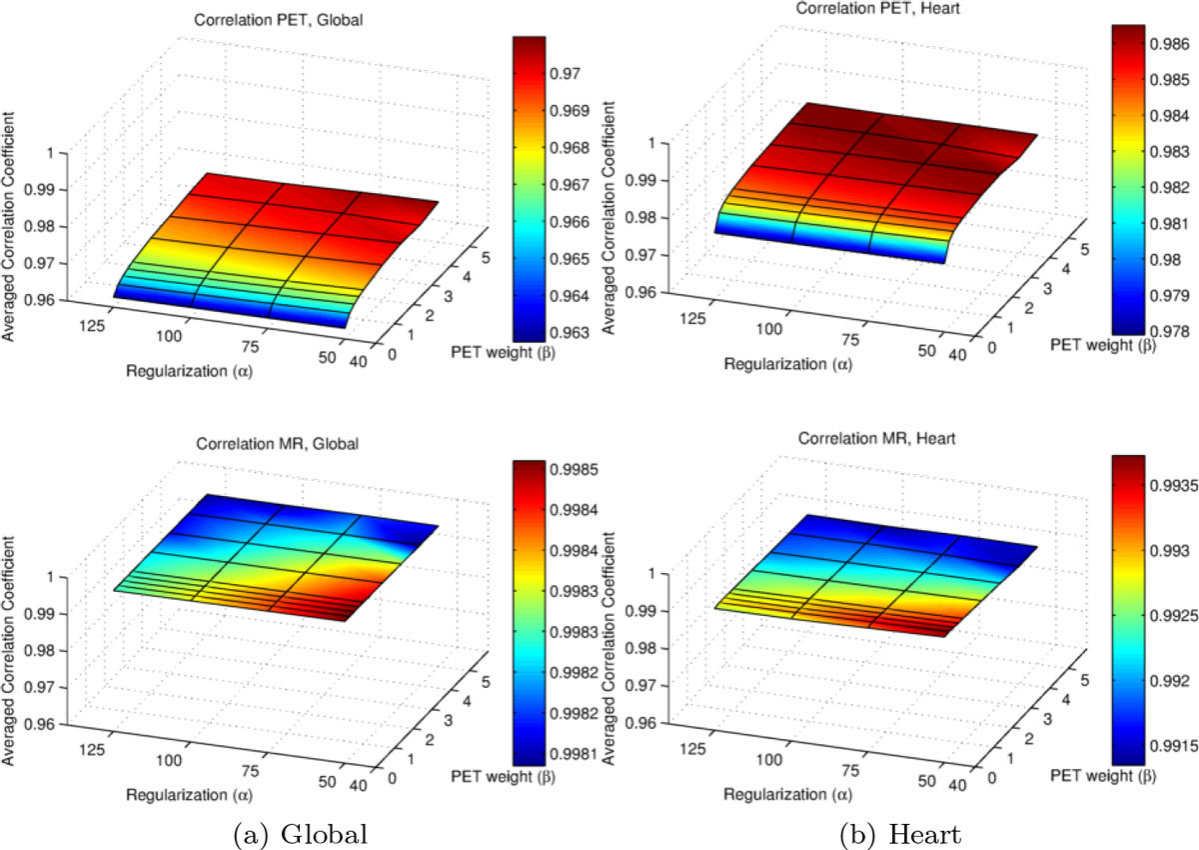
**Averaged Correlation**. The upper plots show the averaged correlation of all PET gates after correction for a range of values of *α *and *β*, the lower plots give the averaged correlation of the MR gates. While the averaged correlation of the PET gates is increased, the correlation of the MR gates is only slightly reduced. For proper comparison, the z-axes are set to the same scaling.

After applying the motion estimates to the input data, we evaluated the average correlation of all corrected PET gates with respect to the target gate. To evaluate if adding PET information to the registration impairs correlation values for MR data, the motion estimates were used to warp the MR data as well. Results are given in Figure 5, for full image data (global) as well as for the heart region. The correlation of the PET data is increased with increasing values of *β *while correlation values of the MR data decrease only slightly.

### Recovered Activity in Lesions

**Figure 6:**
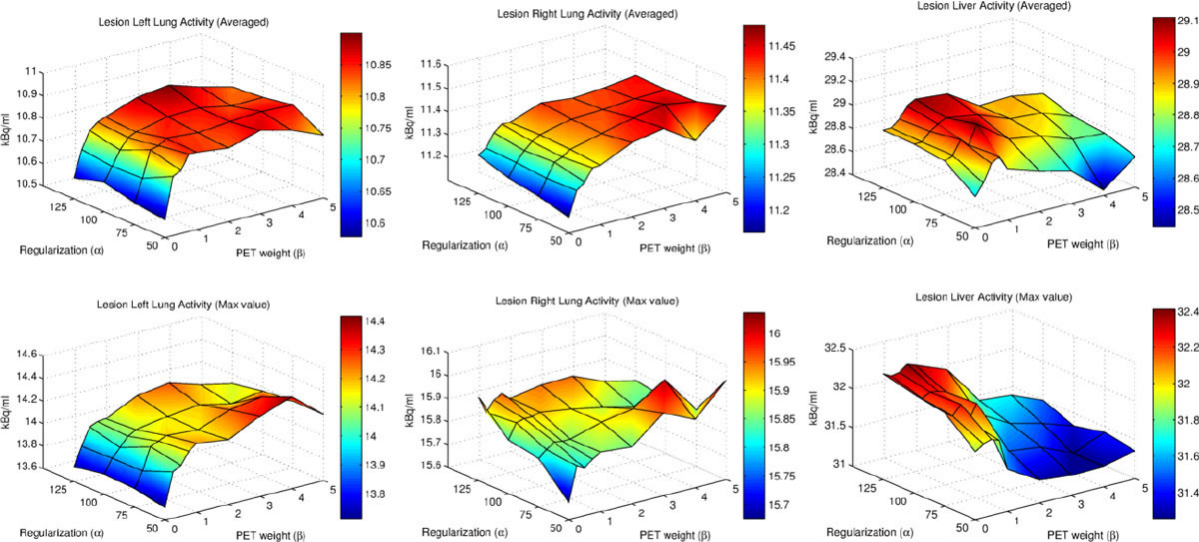
**Lesion Activity Recovery**. Maximum and averaged activities in the added lesions after motion correction using the respective values of *α *and *β*. Generally, addition of PET (moderately increasing values of *β*) lead to a better recovery of the activity.

In Figure 6, evaluation results regarding recovered activity in the three lesions are given as average and maximum activity. Adding information from PET data leads to a better recovery of activity. With very large values of *β*, activity recovery is decreasing again.

## Discussion

Using the joint registration approach, local improvements are observable. For the heart, the lungs, and the lesions (lungs and liver), improvements in terms of registration error (AEE) are achieved. In particular the lesions in the lungs show a large reduction in registration error when PET data are added to the registration. For the liver region, an increase of the AEE is observable. For the area of the lesion added to the liver, though, the AEE is decreased. With the exception of the liver region in toto, those regions that exhibit tracer uptake in PET seem to contribute to a better motion estimation result. Correlation of PET data is improved if PET data is added to the registration, indicating a benefit. Additionally, the lower registration error leads to a slightly better recovery of the activity in the lesions. Globally, a slight increase of the AEE is observable with increasing values of *β*.

The results presented here indicate that a benefit of using motion information from both modalities, MR and PET, is achievable. Certainly, the extent of the benefit will depend on many factors, with one major factor being the image quality of the two image modalities. The amount of remaining motion-induced blurring *within *the individual MR and PET gates will certainly limit the precision to which motion can be estimated. The extent to which PET can contribute will as well depend on the image quality, determined by factors like, e.g., type of injected tracer, injected dose, tracer uptake, and acquisition time. The phantom data used here does not contain artifacts other than noise. Particularly, we did not simulate motion artifacts which are likely to occur during MR acquisitions.

## Conclusion

We have presented a joint registration functional that makes use of motion information derived from PET and MR data simultaneously. In this approach, motion information from both modalities is used. We demonstrated that the proposed method leads to a lower local registration error and better recovery of lesion activity, thus using information from both modalities simultaneously is beneficial regarding motion correction. As a result, clinical scenarios involving lesion quantification might in particular benefit from the proposed method.

In future work, we will evaluate our approach for a broader range of data, including cardiac motion. This will include the addition of mass-preservation [6]. Certainly, the phantom data used in this work does only approximate reality. Thus, we will evaluate the proposed approach on real phantom data.

## Competing interests

The authors declare that they have no competing interests.
